# Development of a Large Pericardial Effusion in a Patient With High-Output Heart Failure and Cardiogenic Shock From an Arteriovenous Fistula

**DOI:** 10.7759/cureus.114572

**Published:** 2026-08-15

**Authors:** Sami Khatib, Chirag Lodha, Erika Lytle, Eric J Basile

**Affiliations:** 1 Department of Internal Medicine, University of South Florida Morsani College of Medicine, Tampa, USA; 2 Department of Cardiology, University of South Florida Morsani College of Medicine, Tampa, USA

**Keywords:** cardiogenic shock, hemodialysis complication, high-flow arteriovenous fistula, high-output heart failure, massive pericardial effusion, pulmonary hypertension

## Abstract

A 58-year-old male with a history of end-stage renal disease on hemodialysis presented with a one-week history of shortness of breath, weakness, and hypotension and was transferred to our institution with concern for cardiogenic shock complicated by severe pulmonary hypertension (HTN) and a large pericardial effusion. Diagnostic workup was concerning for high-output heart failure secondary to his arteriovenous fistula as the underlying etiology, although this was complicated by other potential etiologies, including severe pulmonary HTN and subsequent decompensation from GI hemorrhage. Notably, the pericardial effusion was in a posterior location not amenable to pericardiocentesis. Surgical ligation of the fistula was performed alongside aggressive fluid removal with continuous renal replacement therapy (CRRT), resulting in gradual stabilization of his condition and ultimately complete resolution of his pericardial effusion without the need for percutaneous drainage. This case demonstrates high-output heart failure and cardiogenic shock, an already rare, potentially fatal complication of hemodialysis fistulas, with an associated large pericardial effusion that was successfully managed with surgical ligation of the fistula and CRRT.

## Introduction

Hemodialysis remains a mainstay of treatment for patients with end-stage renal disease (ESRD), and the main long-term access used is the creation of an arteriovenous fistula (AVF). One rare and potentially fatal complication of these fistulas that has been documented in the literature is high-output heart failure and cardiogenic shock [1]. Creation of an AVF results in increased flow from the arterial circulation to the venous circulation, especially in upper extremity AVFs. This leads to increased venous return to the heart, increasing preload drastically and potentially leading to high-output heart failure. While there is not an exact cutoff for normal fistula flow rate, the normal range is typically defined as 0.6-1.5 L/min [2]. In one study, the incidence of high-output heart failure, defined as an inflow rate >1,600 mL/min, a cardiac output >5 L/min, and associated symptoms of heart failure requiring surgical correction of the fistula, was found to be 3.7% (17 of 460 patients) [3].

Patients with ESRD on hemodialysis are also at high risk for pulmonary hypertension (HTN), with studies estimating the prevalence to be between 12% and 49% [4]. In turn, pulmonary HTN is associated with the development of pericardial effusions due to elevated pressures leading to transudation of fluid into the myocardial interstitium and, ultimately, the pericardial space [5]. Thus, there is an association between ESRD, pulmonary HTN, and the development of pericardial effusions. We present a case of a patient with ESRD on long-term hemodialysis via an AVF who developed a large pericardial effusion and cardiogenic shock with a multifactorial etiology, including severe pulmonary HTN and high-output heart failure. Our case was unique due to the large size of the effusion, the posterior location that was not amenable to percutaneous drainage, the severe high-output heart failure and cardiogenic shock, and the resolution of the effusion with fistula ligation, continuous renal replacement therapy (CRRT), and intensive medical management.

This article was previously presented as a poster presentation at the 2026 Florida American College of Physicians (ACP) Chapter Annual Resident and Medical Student Meeting on March 26, 2026.

## Case presentation

A 58-year-old male with a relevant past medical history of renovascular HTN, ESRD status post renal transplantation in 2000 and on hemodialysis since 2017 via a right brachiocephalic AVF created in 2023, and severe pulmonary HTN requiring 3 L of oxygen presented as a transfer to our institution from another hospital with concern for cardiogenic shock complicated by severe pulmonary HTN and a large pericardial effusion. Of note, he had a recent left heart catheterization in 2024 that demonstrated no coronary artery disease, as well as right heart catheterization (RHC) in September 2025 with right atrial pressure of 20 mmHg (N = 2-6 mmHg), mean pulmonary artery pressure (MPAP) of 68 mmHg (N = 8-20 mmHg), and pulmonary capillary wedge pressure (PCWP) of 28 mmHg (N ≤ 15 mmHg) [6]. His other significant medical history included atrial fibrillation, for which he was not on anticoagulation due to a massive GI bleed in 2023, and obstructive sleep apnea on CPAP. His home medication regimen included midodrine 10 mg three times per day for dialysis-associated hypotension and riociguat 1.5 mg three times per day for pulmonary HTN.

He initially presented to another hospital two days prior with a one-week history of progressive shortness of breath, weakness, and hypotension, as well as a near-syncope event. He also reported chest pain while lying supine that improved with leaning forward. He denied any cough, fever, or chills suggestive of an infectious etiology. However, he was also complaining of persistent right-sided neck pain with difficulty lifting his head since a motor vehicle accident two weeks ago in which he fell and scraped his head on the seat. Blood pressure on admission was 67/53 mmHg, and vital signs were otherwise within normal limits. Physical examination was significant for bibasilar crackles, jugular venous distension to the angle of the jaw at 30 degrees, mild 1+ bilateral lower extremity pitting edema, and an irregularly irregular cardiac rhythm.

Laboratory work was significant for a markedly elevated NT-proBNP of 36,500 pg/mL (N < 300 pg/mL). Imaging was significant for a chest X-ray showing diffuse airspace disease and small bilateral pleural effusions and an echocardiogram demonstrating preserved left ventricular ejection fraction with right ventricular overload and a large pericardial effusion without evidence of right ventricular diastolic collapse to suggest impending tamponade. He was transferred due to severe pulmonary HTN and acute-on-chronic right heart failure with preserved ejection fraction to receive ICU-level care, low-dose vasopressors, and possible CRRT for fluid removal. He was transferred on midodrine and droxidopa for chronic hypotension, with riociguat held due to relative hypotension. As for the cervical spine injury, CT and CT angiography imaging demonstrated C4-7 transverse process fractures without evidence of cerebrovascular injury, and no neurosurgical intervention was indicated.

**Figure 1:**
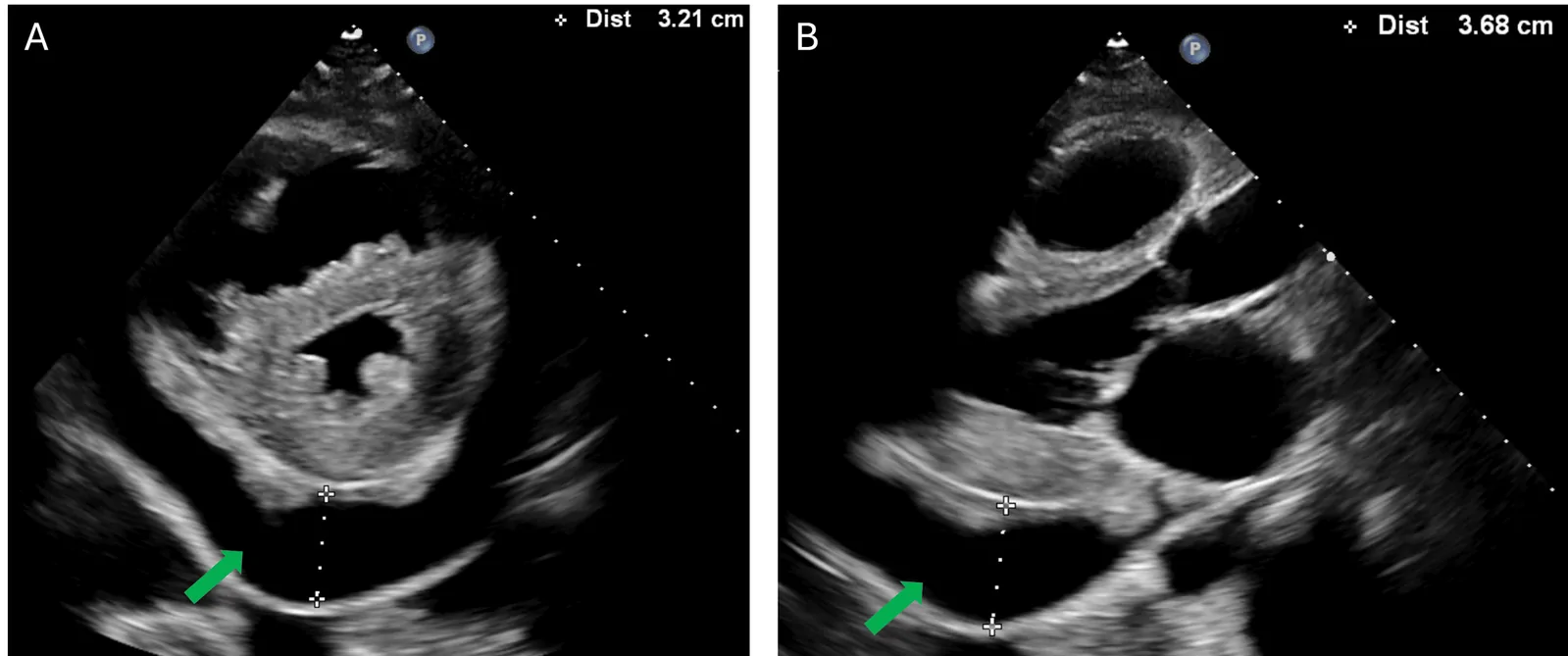
Multiple echocardiographic views of the large pericardial effusion. (A) Parasternal short-axis and (B) parasternal long-axis views of the large pericardial effusion measuring >3 cm (as denoted by the green arrows). The posterior location of the effusion meant there was no window for percutaneous drainage.

Echocardiography done on admission to our hospital demonstrated similar findings (Figure 1). However, because the pericardial effusion was predominantly posterior, there was no window for percutaneous drainage. Furthermore, the posterior location of the effusion, along with his extensive comorbidities, led us to conclude that the potential risks of surgical pericardial window formation outweighed the benefits. RHC showed elevated biventricular filling pressures, severe combined pre- and post-capillary pulmonary HTN with MPAP of 61 mmHg and pulmonary vascular resistance of 5.8 WU (N = 0.3-2.0 WU), and preserved cardiac output of 7 L/min (N = 4-8 L/min) and cardiac index of 3.5 L/min/m² (N = 2.5-4.0 L/min/m²) [6].

Outside Doppler ultrasonography of the fistula from one year prior had shown evidence of high flow at 1.6-2.7 L/min (N = 0.6-1.5 L/min), and repeat evaluation continued to suggest high flow without evidence of focal stenosis, thrombus, or aneurysmal degeneration [2]. Crucially, there was improvement in MPAP upon application of a compression wrap to the fistula. In evaluating the potential etiology of his pericarditis, there was no significant elevation in BUN to suggest uremic pericarditis, no signs or symptoms of infection or leukocytosis, no history or evidence of malignancy, and normal thyroid laboratory results. These findings suggested that ESRD with chronic dialysis use, high-output heart failure, and pulmonary HTN were the most likely underlying causes.

We discussed fistula ligation versus flow reduction via banding but ultimately proceeded with surgical ligation due to the higher likelihood of rapidly and effectively improving his condition through complete closure of the high-flow arteriovenous shunt. Following ligation, MPAP, central venous pressure, and PCWP improved. He was transitioned off CRRT and onto hemodialysis via a left internal jugular vein tunneled dialysis catheter and was stable for transfer to the floor off vasopressors four days later.

However, three days after transfer to the floor, he once again developed worsening hypotension with mean arterial pressures (MAPs) in the 30s, accompanied by altered mental status and blurry vision without any other focal neurologic abnormalities. Administration of midodrine and albumin only improved his MAPs to the 40s, so he was transferred back to the ICU with concern for undifferentiated shock. Lactate was elevated to a maximum of 4.7 mM (N = 0.5-2.2 mM). Repeat echocardiography showed improvement in the size of the pericardial effusion from >3 cm to 1.8 cm without evidence of right ventricular diastolic collapse and with normal respirophasic changes in transvalvular velocities, not suggestive of tamponade, followed by complete resolution of the effusion on repeat echocardiography four days later, making cardiogenic or obstructive shock less likely. In addition, CT angiography of the pulmonary arteries was negative for pulmonary embolism.

Over the following three days, his hemoglobin declined from 11.9 g/dL to 6.8 g/dL (N = 14.1-18.1 g/dL), and he had episodes of melena. Initial esophagogastroduodenoscopy (EGD) showed a marginal gastrojejunal ulcer and a visible vessel that was treated with epinephrine injection, cauterization, and PuraStat. However, he subsequently required blood transfusion, and repeat EGD four days later demonstrated a gastrojejunal anastomosis with extensive ulceration and a large visible vessel without active hemorrhage that were treated with epinephrine and four hemostatic clips. His condition improved over the next few days; his hemoglobin began to recover, and he was stable off vasopressors for transport back to the other hospital.

## Discussion

High-output heart failure and cardiogenic shock are known to be rare but potentially fatal complications of AVFs in patients with ESRD. In a recent systematic review of mixed studies including 18,735 patients, Yasir et al. found that surgical ligation of patent AVFs in renal transplant recipients with high-output heart failure resulted in significant improvement in both objective findings, such as left ventricular mass index, left ventricular end-diastolic diameter, and systemic vascular resistance index, as well as subjective symptoms [1]. In addition, pulmonary HTN was noted to be a common finding in these patients, and AVF ligation resulted in significant improvement in right-sided pressures [1]. Our patient differs from the study population in that he had a history of transplant failure rather than a functioning transplant, and aggressive fluid removal with CRRT and intensive medical management played a significant role in his recovery. Nevertheless, we observed similar findings in our patient following treatment with AVF ligation and CRRT, as there was improvement in his right-sided pressures within a few days post-ligation, and he was clinically stable enough to be transferred to the floor. His subsequent decompensation was found to be due to an acute GI bleed rather than an underlying cardiac etiology, and he stabilized again with management of this bleed.

Interestingly, there is little mention of large pericardial effusions being present in these patients. Sharma et al. described a case of a 15-year-old male with ESRD and a six-year history of hemodialysis who developed pulmonary HTN and a large pericardial effusion [7]. He was treated with pericardiocentesis, aggressive dialysis, and sildenafil acutely, and later sildenafil and bosentan chronically, resulting in some improvement in his signs and symptoms [7]. In contrast, our case was in a significantly older patient with associated heart failure and a pericardial effusion that was successfully managed without drainage. In our case, we were unable to perform a pericardiocentesis due to the posterior location of the fluid, but it improved and then resolved on subsequent echocardiograms following AVF ligation and CRRT without the need for percutaneous drainage.

Pulmonary HTN is a common comorbidity in patients on hemodialysis, with various studies estimating the prevalence to be between 12% and 49%, and it is more prevalent in patients on hemodialysis than in patients on peritoneal dialysis [4]. The exact pathophysiologic mechanism behind this association is unclear, but a number of possible explanations have been studied, including chronic inflammation, excessive pulmonary blood flow, uremia-induced pulmonary vasoconstriction and calcification, microbubbles obstructing the pulmonary microvasculature, or some combination of these factors [4]. In addition, there is a known link between pulmonary HTN and pericardial effusions [5]. Elevated pulmonary artery pressure leads to increased right-sided pressures, leading to increased coronary sinus and Thebesian vein pressure, which ultimately results in transudation of fluid into the myocardial interstitium [5]. Increased right-sided pressures also result in elevation of the pressure in the superior vena cava, which impedes myocardial lymphatic drainage and further worsens myocardial edema [5]. Finally, this myocardial edema results in increased interstitial pressure that forces the fluid into the pericardial space, resulting in the formation of a pericardial effusion [5]. Crucially, the presence of a pericardial effusion in patients with pulmonary HTN has been shown to worsen prognosis, with patients who have larger pericardial effusions or effusions that do not resolve over time having higher mortality rates [5,8-10]. In this case, the presence of a variety of other comorbidities, such as ESRD, chronic dialysis, and right heart failure, in addition to his pulmonary HTN suggests there was likely a multifactorial etiology underlying the pericardial effusion and further complicated management.

## Conclusions

High-output heart failure and cardiogenic shock are rare yet notable complications of AVFs that can be further complicated by severe pulmonary HTN and a large pericardial effusion. Cardiogenic shock from high-output heart failure alone carries a significant mortality risk, and the presence of a large pericardial effusion is another poor prognostic indicator, especially considering that its posterior location made it not amenable to percutaneous drainage. Ligation of the AVF has been demonstrated to be effective in improving heart failure signs and symptoms in these patients, but our case demonstrates that its use in conjunction with aggressive fluid removal via CRRT was followed by resolution of the pericardial effusion without the need for pericardiocentesis. This may have been due to these treatments addressing one of the potential underlying mechanisms for the development of the effusion via improvement in pulmonary HTN. Clinicians should be vigilant for a developing pericardial effusion in AVF-induced high-output heart failure, with treatment being directed toward pericardiocentesis if possible. In addition, banding or ligation of the AVF should be considered, although this must be an individualized decision that considers the severity of the high-output heart failure, whether it responds to more conservative measures, and what other vascular access options are available to the patient for future hemodialysis. Distal AVF placement in patients with a history of high-output heart failure could potentially be considered in those with ESRD requiring dialysis to prevent recurrence of this complication.

## References

[1] Yasir MB, Man RK, Gogikar A, Nanda A, Niharika Janga LS, Sambe HG, Mohamed L (2024). A systematic review exploring the impact of arteriovenous fistula ligature on high-output heart failure in renal transplant recipients. Ann Vasc Surg.

[2] Lok CE, Huber TS, Lee T (2020). KDOQI clinical practice guideline for vascular access: 2019 update. Am J Kidney Dis.

[3] Chemla ES, Morsy M, Anderson L, Whitemore A (2007). ASDIN Original Investigations: Inflow reduction by distalization of anastomosis treats efficiently high-inflow high-cardiac output vascular access for hemodialysis. Semin Dial.

[4] Kosmadakis G, Aguilera D, Carceles O, Da Costa Correia E, Boletis I (2013). Pulmonary hypertension in dialysis patients. Ren Fail.

[5] Stewart RH, Cox CS Jr, Allen SJ, Laine GA (2022). Myocardial edema provides a link between pulmonary arterial hypertension and pericardial effusion. Circulation.

[6] Humbert M, Kovacs G, Hoeper MM (2022). 2022 ESC/ERS Guidelines for the diagnosis and treatment of pulmonary hypertension: developed by the task force for the diagnosis and treatment of pulmonary hypertension of the European Society of Cardiology (ESC) and the European Respiratory Society (ERS). Endorsed by the International Society for Heart and Lung Transplantation (ISHLT) and the European Reference Network on rare respiratory diseases (ERN-LUNG). Eur Heart J.

[7] Sharma S, Kirpalani AL, Kulkarni A (2010). Severe pulmonary hypertension in a young patient with end-stage renal disease on chronic hemodialysis. Ann Pediatr Cardiol.

[8] Batal O, Dardari Z, Costabile C, Gorcsan J, Arena VC, Mathier MA (2015). Prognostic value of pericardial effusion on serial echocardiograms in pulmonary arterial hypertension. Echocardiography.

[9] De Filippo O, Gatti P, Rettegno S (2019). Is pericardial effusion a negative prognostic marker? Meta-analysis of outcomes of pericardial effusion. J Cardiovasc Med (Hagerstown).

[10] Fenstad ER, Le RJ, Sinak LJ (2013). Pericardial effusions in pulmonary arterial hypertension: characteristics, prognosis, and role of drainage. Chest.

